# Supplementary material for: Further Advance of Gambierdiscus Species in the Canary Islands, with the First Report of Gambierdiscus belizeanus
Source: Toxins (Basel). 2020 Oct 31;12(11):692. doi: 10.3390/toxins12110692 (PMC7693352; doi:10.3390/toxins12110692)
Supplement: Supplementary file 1 [file toxins-12-00692-s001.zip › toxins-958273 supp/toxins-958273-table s3.docx]

**Supplementary Materials:** **Further Information of *Gambierdiscus* Species in the Canary Islands, with the First Report of *Gambierdiscus belizeanus***

**Àngels Tudó, Greta Gaiani, Maria Rey Varela, Takeshi Tsumuraya, Karl B. Andree, Margarita Fernández-Tejedor, Mònica Campàs and Jorge Diogène**

**Table S3.** Cell viability and CTX-like toxicity after the exposure of neuro-2a cells to *Gambierdiscus* spp. extracts with (O/V^+^) and without (O/V^−^) ouabain and veratridine treatment.

|  |  |  | **% neuro-2a cells viability** | |  |  | |
| --- | --- | --- | --- | --- | --- | --- | --- |
| **Strain code** | **Species** | **Dose (cells equiv. mL^−1^)** | **O/V^−^**  **Mean ± SD**  ***N* = 3** | **O/V^+^**  **Mean ± SD**  ***N* = 3** | **CTX1 (pg mL^-1^) mean ± SD**  ***N* = 3** | | **Toxin content (fg CTX1B equiv. cell^-1^)**  **Mean ± SD** |
| IRTA-SMM-17-004 | *G. australes* | 3.38 | 99 ± 1.4 | 64.9 ± 6.8 | 0.7 ± 0.1 | 205.1 ± 34.5 | |
| IRTA-SMM-17-006 | *G. australes* | 17.75 | 107.1 ± 1.3 | 45.1 ± 14.7 | 2.3 ± 1.5 | 127.7 ± 85 | |
| IRTA-SMM-16-288 | *G. australes* | 25.00 | 121.7 ± 8.9 | 41.3 ± 16.3 | 2.7 ± 1.9 | 106.1 ± 75.3 | |
| IRTA-SMM-16-290 | *G. australes* | 55.80 | 114.5 ± 2.3 | 39.7 ± 13.1 | 2.6 ± 1.2 | 46.1 ± 22.2 | |
| IRTA-SMM-16-292 | *G. australes* | 25.00 | 110.4 ± 2.7 | 61.9 ± 6.3 | 1 ± 0.3 | 39.7 ± 10.5 | |
| IRTA-SMM-16-286 | *G. australes* | 38.25 | 115.3 ± 10.4 | 33.7 ± 4.8 | 1.3 ± 0.2 | 33.6 ± 6.5 | |
| IRTA-SMM-16-293 | *G. australes* | 21.00 | 112.1 ± 2.4 | 69.5 ± 5.5 | 0.7 ± 0.2 | 32.7 ± 10 | |
| IRTA-SMM-17-007 | *G. australes* | 72.45 | 97.5 ± 1.5 | 68.7 ± 3.3 | 1.1 ± 0.1 | 15.8 ± 1.7 | |
| IRTA-SMM-17-002 | *G. australes* | 5.80 | 87.8 ± 5.3 | 34.4 ± 1.5 | 2.7 ± 0.2 | 452.6 ± 23.2 | |
| IRTA-SMM-17-103 | *G. australes* | 40.30 | 111.5 ± 5 | 37.8 ± 9.4 | 4.8 ± 1.2 | 118.2 ± 30.3 | |
| IRTA-SMM-17-107 | *G. australes* | 127.00 | 112.3 ± 2.9 | 50.1 ± 6.3 | 1.5 ± 0.3 | 12.2 ± 2.1 | |
| IRTA-SMM-17-112 | *G. australes* | 201.00 | 109.3 ± 3 | 47.1 ± 10.3 | 0.4 ± 0.1 | 1.9 ± 0.6 | |
| IRTA-SMM-17-106 | *G. australes* | 82.25 | 111.2 ± 2.2 | 77 ± 2.6 | 0.1 ± 0 | 1.7 ± 0.1 | |
| IRTA-SMM-17-358 | *G. australes* | 7.25 | 107.5 ± 7.2 | 47.7 ± 6.4 | 1 ± 0.1 | 138.9 ± 17.7 | |
| IRTA-SMM-17-291 | *G. australes* | 10.75 | 106 ± 5.8 | 34.4 ± 8.7 | 0.9 ± 0.2 | 82.8 ± 22.2 | |
| IRTA-SMM-17-316 | *G. australes* | 7.25 | 115.6 ± 2.4 | 62.5 ± 3.1 | 0.4 ± 0.1 | 51.5 ± 6.9 | |
| IRTA-SMM-17-307 | *G. australes* | 15.75 | 112.4 ± 8 | 51.9 ± 10.2 | 0.6 ± 0.2 | 37.3 ± 12.6 | |
| IRTA-SMM-17-436 | *G. australes* | 9.50 | 119.6 ± 1.3 | 76 ± 7 | 0.9 ± 0.2 | 98.6 ± 25.4 | |
| IRTA-SMM-17-393 | *G. australes* | 21.00 | 115.4 ± 3.9 | 55.7 ± 16 | 0.9 ± 0.2 | 44.6 ± 11.5 | |
| IRTA-SMM-17-344 | *G. australes* | 45.50 | 109.5 ± 10.8 | 29.2 ± 0.1 | 1.9 ± 0.0 | 41.2 ± 0.1 | |
| IRTA-SMM-17-335 | *G. australes* | 15.00 | 112.8 ± 3.4 | 56.6 ± 9 | 0.4 ± 0.1 | 29.1 ± 8.6 | |
| IRTA-SMM-17-287 | *G. australes* | 110.00 | 114.3 ± 4.8 | 52.2 ± 6.3 | 1.2 ± 0.3 | 11.3 ± 2.3 | |
| IRTA-SMM-17-288 | *G. australes* | 116.22 | 111.9 ± 5.2 | 50.8 ± 23.3 | 0.7 ± 0.4 | 5.7 ± 3.8 | |
| IRTA-SMM-17-389 | *G. australes* | 2.09 | 110.1 ± 3.4 | 53.5 ± 3.5 | 0.5 ± 0.1 | 226.5 ± 24.6 | |
| IRTA-SMM-17-324 | *G. australes* | 9.06 | 104.9 ± 4.1 | 31.9 ± 1 | 1.5 ± 0.2 | 160.4 ± 17.2 | |
| IRTA-SMM-17-418 | *G. australes* | 13.13 | 102.2 ± 2.3 | 45.2 ± 3.9 | 0.9 ± 0.1 | 68.3 ± 9.5 | |
| IRTA-SMM-17-321 | *G. australes* | 75.00 | 103.4 ± 4.3 | 44.1 ± 5.2 | 2.4 ± 1.1 | 31.9 ± 15 | |
| IRTA-SMM-17-425 | *G. australes* | 60.50 | 110.4 ± 3.8 | 31.5 ± 4.8 | 1.7 ± 0.2 | 27.8 ± 3.3 | |
| IRTA-SMM-17-327 | *G. australes* | 40.88 | 111.8 ± 2.9 | 67.7 ± 0.9 | 0.3 ± 0 | 7.2 ± 0.3 | |
| IRTA-SMM-17-421 | *G. belizeanus* | 164.50 | 96.3 ± 11.7 | 60.4 ± 0.5 | 0.9 ± 0 | 5.6 ± 0.1 | |
| IRTA-SMM-17-003 | *G. caribaeus* | 6808.00 | 103.6 ± 5.3 | 100 ± 7.4 |  |  | |
| IRTA-SMM-17-001 | *G. excentricus* | 2.50 | 124.2 ± 9.3 | 68.2 ± 7.8 | 2.9 ± 0.5 | 1149.3 ± 212.3 | |
| IRTA-SMM-17-126 | *G. excentricus* | 4.72 | 93.3 ± 1.7 | 44.5 ± 4.7 | 1.1 ± 0.1 | 226.7 ± 22.1 | |
| IRTA-SMM-17-128 | *G. excentricus* | 47.50 | 96.9 ± 10.6 | 65.8 ± 11 | 0.5 ± 0.1 | 9.5 ± 2.6 | |
| IRTA-SMM-17-404 | *G. excentricus* | 0.22 | 97.2 ± 2.5 | 68.7 ± 6.3 | 0.3 ± 0.1 | 1257.6 ± 319.3 | |
| IRTA-SMM-17-405 | *G. excentricus* | 0.63 | 86.2 ± 3.7 | 40.4 ± 6.8 | 0.7 ± 0.2 | 1153.4 ± 238.8 | |
| IRTA-SMM-17-386 | *G. excentricus* | 31.33 | 88.7 ± 3.6 | 69.2 ± 7.1 | 0.4 ± 0.1 | 12.8 ± 2.8 | |
| IRTA-SMM-17-429 | *G. excentricus* | 0.45 | 103.3 ± 1.2 | 49.8 ± 16.5 | 0.7 ± 0.3 | 1525.9 ± 634.1 | |
| IRTA-SMM-17-432 | *G. excentricus* | 0.44 | 109.1 ± 5.7 | 66.1 ± 5.1 | 0.4 ± 0.1 | 962.1 ± 154.7 | |
| IRTA-SMM-17-413 | *G. excentricus* | 30.13 | 109.4 ± 1 | 54.2 ± 8.1 | 0.5 ± 0.2 | 18.1 ± 5.7 | |
| IRTA-SMM-17-330 | *G. excentricus* | 0.30 | 84.3 ± 1 | 60.7 ± 5.7 | 0.8 ± 0.1 | 2566.7 ± 333.3 | |
